# Supplementary material for: Effect of Estrogen on Sirt1 Signaling in Human Macrophages
Source: Int J Mol Sci. 2025 Sep 5;26(17):8670. doi: 10.3390/ijms26178670 (PMC12428810; doi:10.3390/ijms26178670)
Supplement: Supplementary file 1 [file ijms-26-08670-s001.zip › ijms-3820967-Supplementary Figures.pdf]

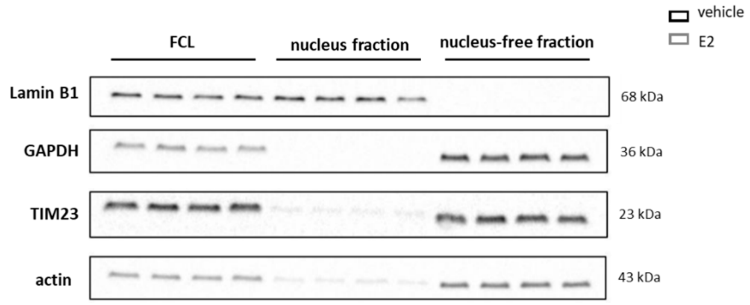

Figure S1. Analysis of the purity of the nuclear fraction isolated from THP-1 cells. Western blot analysis of Lamin B1, GAPDH, TIM23, and actin performed with the nuclear fraction, nuclei-free fraction, and full cell lysate of THP1 cells.

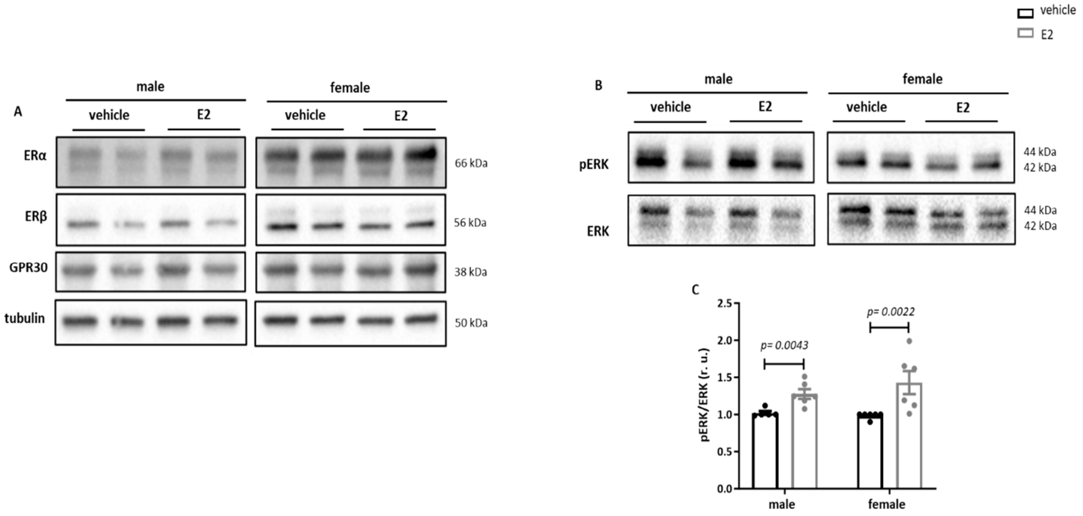

Figure S2. Assay of ER expression and activity in human monocyte-derived macrophages. Western blot analysis of (**A**) ERα, ERβ, and GPR30 and (**B**,**C**) pERK/ERK performed with lysates of male and female primary human M1-macrophages after 24 h treatment with E2 (10 nmol/L). Data are shown as the means ± SEM (n = 5–6). All data were normalized to the corresponding control and expressed in relative units (r.u.). M1 macrophages: 10 ng/mL LPS and 10 ng/mL IFN- γ treatment for 24 h.
