# Supplementary material for: Effect of Estrogen on Sirt1 Signaling in Human Macrophages
Source: Int J Mol Sci. 2025 Sep 5;26(17):8670. doi: 10.3390/ijms26178670 (PMC12428810; doi:10.3390/ijms26178670)
Supplement: Supplementary file 1 [file ijms-26-08670-s001.zip › ijms-3820967-Supplementary Table.pdf]

Table S1. Characterization of donors.

	Men (n= 14)	Women (n= 13)
Age	28.0 (8.0)	22.5 (3.0)
BMI	24.0 (2.0)	21.0 (4.0)
Smoking status:		
• Current smoker	0%	0%
• Former smoker	9%	9%
• Never smoker	82%	91%
• Unknown	9%	0%
Physical activity:		
• < 1 hour	9%	10%
• 1 – 3-hours	36%	50%
• > 3 hours	27%	30%
• Unknown	27%	10

Age and BMI data are shown as median and IQR. Smoking status and physical activity data are shown as percentage. IQR: interquartile range.
